# Case series discussion of cardiac and vascular events following carfilzomib treatment: possible mechanism, screening, and monitoring

**DOI:** 10.1186/1471-2407-14-915

**Published:** 2014-12-04

**Authors:** Ajai Chari, Daher Hajje

**Affiliations:** Mt Sinai School of Medicine, 1 Gustave Levy Place, Box 1185, New York, NY USA; New York University School of Medicine, 500 1st Avenue, New York, NY USA

**Keywords:** Carfilzomib, Proteasome inhibitor, Cardiac toxicity, Vascular toxicity, Multiple myeloma

## Abstract

**Background:**

Carfilzomib is a selective proteasome inhibitor approved in the United States in 2012 for the treatment of relapsed and refractory multiple myeloma. Although cardiopulmonary and vascular events have been reported infrequently, they can be potentially serious complications, and their incidence and pathophysiology following carfilzomib treatment remain poorly characterized in a real-world patient population.

**Methods:**

We retrospectively reviewed the records of 67 patients with relapsed and/or refractory multiple myeloma treated at our institution.

**Results:**

We describe 12 patients who experienced cardiac or vascular-related adverse events subsequent to carfilzomib-based treatment (median age, 59 years [range, 49–77]). Nine patients had prior autologous stem cell transplant, and three had prior anthracycline exposure. Detailed case reports are provided for five representative patients: (1) systemic hypertension in a 65-year-old Caucasian female with a history of hypertension, hypothyroidism, and stage III chronic kidney disease; (2) pulmonary hypertension in a 72-year-old Caucasian male with a history of recurrent respiratory infections and chronic right lower extremity deep venous thrombosis; (3) acute renal insufficiency with increased blood pressure in a 50-year-old Caucasian male with a history of hypertension and stage IV chronic kidney disease; (4) heart failure in a 64-year-old African American female with a history of hypertension; and (5) dyspnea and lung disease in a 58-year-old Asian American male with a history significant for hepatitis B virus infection.

**Conclusions:**

While cardiac and vascular-related adverse events were reported in patients with relapsed and/or refractory multiple myeloma who were treated with carfilzomib, most patients had a history of the specific cardiac or vascular adverse event they exhibited and demonstrated an improvement or resolution in symptoms after the discontinuation of therapy. Appropriate screening and monitoring could potentially allow at-risk patients to benefit fully from treatment with carfilzomib.

## Background

The median overall survival for patients with multiple myeloma (MM) has progressively increased from 22.5 months for patients diagnosed before 1996 to 66.1 months for those diagnosed after 2005 [1]. This is due, in part, to the development and increased usage of proteasome inhibitors such as the boronate-based bortezomib. More recently, the epoxyketone-based proteasome inhibitor carfilzomib was approved in the United States for patients with MM who have received two or more prior therapies, including bortezomib and an immunomodulatory agent, and have had progression of disease within 60 days of completion of their last treatment [2, 3]. In this relapsed and refractory MM population, carfilzomib appears to have a hematologic toxicity profile similar to that of bortezomib, but while neuropathy is a dose-limiting toxicity of the latter agent, this adverse event is rarely seen with carfilzomib treatment. While receiving carfilzomib, the non-hematologic toxicities of fatigue, nausea, dyspnea, diarrhea, pyrexia, headache, upper respiratory infection, and increased blood creatinine were reported in 25–46% (any grade) of patients. Other, less common but significant toxicities included cardiac failure (reported in 7% of patients) and pulmonary arterial hypertension (reported in 2% of patients) [3]. However, an analysis of 526 patients from four phase II studies found that only 1% of carfilzomib-treated patients received a dose reduction and 4% discontinued treatment due to a cardiac event [4]. The same analysis demonstrated that the majority of dyspnea events reported on study were isolated grade 1 or 2 events and rarely resulted in drug discontinuation [4].

While cardiopulmonary and vascular events have the potential to be serious complications, their incidence and pathophysiology following carfilzomib treatment are, as of yet, unclear in a real-world patient population, as these findings only included those patients who were healthy enough to enroll in a clinical trial. In addition, detailed characterizations of the development, management, and outcomes of cardiovascular events following carfilzomib treatment have not been published to date. We report here representative case examples from Mount Sinai Medical Center of carfilzomib-treated patients with MM who developed cardiac or vascular-related events. We then present preventative and monitoring approaches from our institution and conclude by discussing possible mechanisms underlying these events.

### Carfilzomib pharmacology, efficacy, and safety

Carfilzomib is an irreversible covalent inhibitor of the chymotrypsin-like activity of the constitutive proteasome and immunoproteasome [5, 6]. Carfilzomib is considered highly selective for the chymotrypsin-like activity of the proteasome and inhibits its action with comparable potency relative to bortezomib. However, carfilzomib is relatively more selective for the chymotrypsin-like site, as bortezomib inhibits the trypsin-like and caspase-like activity of the proteasome with greater potency [7].

In patients with solid tumors or hematologic malignancies, carfilzomib elimination was rapid, with a mean elimination half-life of less than 30 min [8–11]. Maximum plasma concentrations (C_max_) and area under the curve were found to increase with dose, although the increase was not dose-proportional [8, 11]. Clearance is thought to be through multiple extrahepatic clearance pathways [8, 11].

The accelerated approval of carfilzomib in the United States was based on the efficacy and safety data from the phase II PX-171-003-A0 study (N =46) [12], as well as safety data from PX-171-003-A1 (N =266) [2], PX-171-004 (N =129) [13], and PX-171-005 (N =50) [10] studies. Carfilzomib is administered intravenously over 2–10 min as a single agent at a starting dose of 20 mg/m^2^ in cycle 1 on days 1, 2, 8, 9, 15, and 16 of a 28-day cycle, followed by escalation to a target dose of 27 mg/m^2^ during cycle 2 and beyond [3]. In the PX-171-003-A1 study of relapsed and refractory MM, this schedule was associated with an objective response rate (ORR) of 24%, median progression-free survival (PFS) of 3.7 months, and a favorable safety profile.

Preclinical studies of carfilzomib have demonstrated that the *in vivo* potency of carfilzomib is likely a consequence of total dose administered and not maximum concentration (C_max_) [14]. Combined with findings in bortezomib, which have suggested that peripheral neuropathy risk may be associated with higher C_max_ values [15], longer carfilzomib infusion times have been examined in the clinic under the hypothesis that this would allow for the administration of higher doses of carfilzomib with reduced toxicity compared with shorter infusion times. The phase Ib PX-171-007 trial [16] in relapsed and/or refractory MM administered carfilzomib as an infusion over 30 min, resulting in an approximately three-fold lower C_max_ and similar area under the curve relative to administration as a 2–10-min infusion. At the maximum tolerated dose of 56 mg/m^2^, improved efficacy (ORR of 60%) was observed relative to the recommended dose (27 mg/m^2^). The higher dose of carfilzomib was associated with inhibition of all three immunoproteasome subunits and superior inhibition of chymotrypsin-like proteasome activity in peripheral blood mononuclear cells compared with 20 mg/m^2^ carfilzomib (≥95% vs. 80%, respectively). In this study, grade 3 or 4 hypertension was reported in 13% of patients.

Similar efficacy results were seen in the IST-CAR-512 study, which examined 56 mg/m^2^ carfilzomib administered as a 30-min infusion in 41 patients [17], and reported an ORR of 53% and median PFS of 7.6 months. The authors also reported a number of grade 3 and 4 cardiopulmonary adverse events, including hypertension in eight patients (20%)— also seen in PX-171-007—as well as pulmonary edema/congestive heart failure (CHF) in four patients (10%) and left ventricular systolic dysfunction in two patents (5%). It must be noted, however, that patients received up to 500 mL of hydration before and after each dose of carfilzomib (up to 6 L per cycle). In addition, with a median of five lines of prior therapy, including 40% with an Eastern Cooperative Oncology Group performance status of 2, a significant number of these patients had also undergone allogeneic transplantation and, therefore, likely had an increased risk of cardiovascular complications [18]. Concomitant medications (e.g., calcineurin inhibitors) may also have played a role in the development of hypertension. Therefore, both PX-171-007 and IST-CAR-512 suggest that a higher dose of carfilzomib results in better efficacy than the approved 27-mg/m^2^ dose. However, there is also an increased rate of cardiovascular adverse events, although these events differed in nature and frequency between the two studies.

## Methods

In the analysis presented here, we retrospectively reviewed the electronic medical records of 67 consecutive patients with relapsed and/or refractory MM from August 2012 to December 2012 who were treated with carfilzomib on a clinical trial, on the compassionate use program (NCT01410500), or using commercial supply after drug approval. Patients were typically treated with carfilzomib and dexamethasone, although a few received additional antimyeloma agents in combination with carfilzomib. All patients reviewed received 20 mg/m^2^ carfilzomib in cycle 1 on days 1 and 2 only. Thereafter, patients received 27 mg/m^2^ on days 8, 9, 15, and 16 of cycle 1 (28-day cycle). During cycles 2 and beyond, 27 mg/m^2^ was administered on days 1, 2, 8, 9, 15, and 16. This schedule differs from the approved dose/schedule, where dose escalation to 27 mg/m^2^ occurs at the beginning of the cycle 2, rather than on cycle 1, day 8 [3]. Carfilzomib was administered intravenously over 2–10 min in all patients. Adverse events were assessed using the National Cancer Institute Common Terminology Criteria for Adverse Events (CTCAE), version 4.0. This analysis was approved by the Mount Sinai School of Medicine’s institutional review board. Written informed consent was obtained from all patients, or next of kin in the case of deceased patients, for publication in this Case series. Copies of the written consent are available for review by the Editor of this journal.

## Results

From the 67 charts that were reviewed, 12 patients were identified as having a non-hematologic adverse event of grade 3 or higher (per CTCAE) that was possibly due to treatment with carfilzomib, as suggested based on the treatment-emergent nature of these adverse events and because there were no other new drug exposures in these patients. Baseline characteristics are shown in Table 1. Nine patients had prior autologous stem cell transplant, and no patients had prior allogeneic transplant. Three patients had prior anthracycline exposure. No patients identified in this analysis had a prior history suggestive of amyloidosis; however, many of the patients had a prior history of the specific cardiac or vascular-related adverse event that they exhibited (Table 2). Next we review in detail five representative cases.

**Table 1:** **Baseline patient and disease characteristics in carfilzomib-treated patients reporting a cardiac or vascular-related adverse event**

	Patients N = 12
Age, median years (range)	59 (49–77)
Male sex, %	50
Durie-Salmon stage at diagnosis	
IA	3
IIA	1
IIIA	7
IIIB	1
ISS stage at diagnosis	
I	0
II	3
III	3
Unknown	6
Lines of previous anti-MM therapy, median	5
Prior bortezomib, n (%)	11 (92)
Prior autologous stem cell transplant, n (%)	9 (75)
Prior anthracycline, n (%)	3 (25)

ISS, International Staging System; MM, multiple myeloma.

**Table 2:** **Incidence of cardiac or vascular**-**related adverse events**

Adverse event of interest	Patients reporting grade ≥3 adverse event,n	Patients reporting grade≥3 adverse event with history of the adverse event,n
Hypertension	5	4
Congestive heart failure	5	3
Pulmonary hypertension	2	2
Lung disease	1	0
Renal failure	6	6

### Case 1: systemic hypertension

A 64-year-old Caucasian female with immunoglobulin (Ig) A kappa MM, Durie-Salmon (DS) stage IIA, and unknown International Staging System (ISS) stage at diagnosis in 2000 had a medical history significant for hypertension, hypothyroidism, and stage III chronic kidney disease. Eleven years after her MM diagnosis, after progressing on seven lines of therapy (most recently while receiving pomalidomide), the patient started carfilzomib 20 mg/m^2^ on days 1 and 2. Over the next 7 days, she had mildly increased blood pressure (grade 2) but returned to a baseline hypertensive state of approximately 150/80 mmHg without intervention. Cycle 2 was delayed owing to hospitalization for urosepsis and back pain from lytic vertebral disease. Upon resolution of these adverse events, carfilzomib was resumed at 27 mg/m^2^. At 30 min post infusion (after a carfilzomib cumulative dose of 175 mg/m^2^), she was noted to be tachycardic to 140 bpm with a blood pressure of 198/102 mmHg. She responded well to treatment with hydralazine 10 mg orally (PO) as needed (PRN), in addition to her baseline regimen of amlodipine 10 mg PO once daily (QD) and carvedilol 20 mg QD. The next day, carfilzomib infusion was well tolerated when both pre- and post-intravenous hydration were omitted. The following day, however, she became persistently hypertensive to a maximum of 219/110 mmHg (grade 3), which responded once again to hydralazine PRN. She completed cycles 2 and 3 with stable hemodynamics but, unfortunately, succumbed to complications of *Clostridium difficile* colitis 90 days later. The patient received a carfilzomib cumulative dose of 418 mg/m^2^.

### Case 2: pulmonary hypertension

A 72-year-old Caucasian male with IgG kappa MM, DS stage IIIA, and unknown ISS stage at diagnosis in 2000 had a history of recurrent respiratory infections that prompted maintenance intravenous Ig therapy and a history of chronic right lower extremity deep venous thrombosis (DVT) off anticoagulation due to persistent thrombocytopenia (platelet count was <50,000/μL due to bone marrow involvement). Twelve years after diagnosis, after progressing on eight lines of therapy, the patient started single-agent carfilzomib and demonstrated disease progression after five cycles. The patient then continued carfilzomib with the addition of oral cyclophosphamide, thalidomide, and prednisone (CCTP). After receiving carfilzomib infusion on cycle 3, day 9 of CCTP, the patient was hospitalized the following day for new-onset cough, shortness of breath, and lower extremity edema in the setting of a right lower lobe *Haemophilus influenzae* pneumonia. Inpatient workup was negative for acute DVT and pulmonary embolus and showed significant levels of brain natriuretic peptide (BNP; 902 pg/mL). Transthoracic echocardiogram revealed moderate pulmonary arterial hypertension (peak right ventricular systolic pressure [RVSP] of 55 mmHg and a gradient of 10 mmHg), worsened tricuspid regurgitation (TR), and an increase in right ventricular (RV) dilatation concomitant with a decrease in function. In comparison, the patient had a 10-block exercise tolerance at baseline prior to starting carfilzomib, and a screening echo revealed a peak RVSP of 35 mmHg with a gradient of 5 mmHg, minimal TR, and normal RV size and function. Therefore, right-sided CHF as a result of worsening pulmonary hypertension was considered the likely diagnosis. The cumulative dose of carfilzomib prior to onset of this episode was 1255 mg/m^2^. Six days after being admitted, the patient was successfully diuresed and discharged to home to complete a course of antibiotics. The patient then elected home hospice.

### Case 3: acute renal insufficiency with increased blood pressure

A 50-year-old Caucasian male with lambda light chain MM, DS stage IIIA, and ISS stage III at diagnosis in 2000 had a medical history significant for hypertension and stage IV chronic kidney disease, with a baseline creatinine level of 4 mg/dL. Eleven years after diagnosis, after progressing on seven lines of therapy (most recently while taking bortezomib, dexamethasone, thalidomide, cisplatin, doxorubicin, cyclophosphamide, and etoposide [VDT-(P)ACE] therapy without cisplatinum), the patient started carfilzomib through a compassionate use program. Routine chemistries collected following infusion of 20 mg/m^2^ carfilzomib on cycle 1, day 1, revealed an elevation in the patient’s creatinine level to 5.54 mg/dL, which then steadily decreased to 4.54 mg/dL just prior to cycle 1, day 8. Post infusion on cycle 1, day 9, the patient’s creatinine level rose to 5.64 mg/dL and increased to 7.30 mg/dL the following day. Five days following the last infusion (after a cumulative carfilzomib dose of 94 mg/m^2^), the patient was admitted for acute-on-chronic, non-oliguric renal failure with a creatinine level of 10.59 mg/dL with complaints of dizziness and weakness. At the time of admission, his blood pressure was 180/100 mmHg compared with a baseline blood pressure of 120/80 mmHg while taking valsartan. There was no history of vomiting or diarrhea, and he was euvolemic upon examination. Doses of carfilzomib on days 15 and 16 were delayed, and he was treated with intravenous fluid with bicarbonate, labetalol, and sevelamer. He was discharged home with a creatinine level of 6.8 mg/dL. Seven days after discharge, he resumed treatment with carfilzomib (cycle 2, day 1) at a reduced dose of 20 mg/m^2^. His renal function was not subsequently affected by further treatment and returned to baseline 120 days later, after the patient had completed five cycles of carfilzomib treatment (a cumulative dose of 541 mg/m^2^) and was taken off the study to receive an allogeneic stem cell transplant.

### Case 4: Heart failure

A 64-year-old African American female with IgA kappa MM, DS Stage IIA, and unknown ISS stage at diagnosis in 2006 had a medical history of hypertension. The patient had an ejection fraction (EF) of 69% by multi-gated acquisition scan prior to receiving bortezomib, dexamethasone, and 30 mg/m^2^ liposomal doxorubicin as a fifth-line regimen. A repeat transthoracic echocardiogram showed an EF of 50% after five cycles of this regimen (and a cumulative liposomal doxorubicin dose of 150 mg/m^2^). After receiving two additional lines of therapy consisting of salvage high-dose melphalan with autologous stem cell transplant with bortezomib maintenance and dexamethasone, cyclophosphamide, etoposide, and cisplatin (DCEP) chemotherapy, she was enrolled into the carfilzomib compassionate use program. Her last known EF was 40–45% as assessed 2 years prior to starting carfilzomib, and she was New York Heart Association (NYHA) class I. She developed progressively worsening dyspnea 6 days after completing cycle 2, day 9 of carfilzomib treatment. The patient was admitted to a local hospital, where she was found to have acute biventricular CHF with a left ventricular EF of 14% and a BNP level of 34,000 pg/mL. The patient had received carfilzomib along with 20 mg of dexamethasone intravenously and 250 mL of pre- and post-infusion intravenous hydration with normal saline. The cumulative dose of carfilzomib prior to onset of CHF was 228 mg/m^2^. Doses on cycle 2, days 15 and 16, were omitted. Given the absence of alternative anti-myeloma therapies, cycle 3 was resumed once the CHF resolved, with a reduced dose of 20 mg/m^2^ carfilzomib, reduced dexamethasone (10 mg prior to each dose), and no intravenous hydration. On cycle 3, day 8, the patient again developed dyspnea, at which time repeat echocardiogram confirmed EF of 15–20%. The patient concluded therapy on cycle 4, day 1, at 20 mg/m^2^ (after a cumulative carfilzomib dose of 362 mg/m^2^) and was withdrawn from the study. She was transitioned to hospice, where she died 120 days later.

Among the five patients who developed CHF, excluding the patient just described, two others also had prior anthracycline exposure. The second patient had received liposomal doxorubicin approximately 3 years prior to starting carfilzomib treatment, had a baseline left ventricular EF of 50%, and was NYHA class 1 prior to receiving carfilzomib as the eighth line of therapy. The patient then developed CHF with an EF of 23% after cycle 2, and carfilzomib was discontinued after day 1 of cycle 4, by which point a cumulative dose of 396 mg/m^2^ had been administered. After four monthly cycles of treatment with bortezomib and bendamustine, a repeat echocardiogram demonstrated an improvement in EF to 53%. However, due to disease progression, the patient elected hospice. The third patient had previously received adriamycin as part of bortezomib-based induction therapy and had a prior history of CHF while receiving celecoxib, which had resolved to normal EF. The patient achieved a minimal response during 12 cycles of carfilzomib 20 mg/m^2^ (for a cumulative dose of 1160 mg/m^2^) as part of the PX-171-003-A0 study and, 14 months later, received carfilzomib through a single-patient compassionate use program for a total of 37 cycles and a cumulative dose of 5746 mg/m^2^, but was noted to have CHF with an EF of 15% after cycle 30.

### Case 5: dyspnea and lung disease

A 58-year-old Asian American male with IgG kappa MM, DS stage III, and ISS stage II at diagnosis in 2010 had a medical history significant for hepatitis B virus (HepB) infection with clearance of HepB DNA while on maintenance tenofovir. Two years after diagnosis, after progressing on three lines of therapy (having most recently received bortezomib and dexamethasone), the patient started treatment with carfilzomib, dexamethasone, and cyclophosphamide. During infusion of 27 mg/m^2^ carfilzomib on cycle 2, day 8, he was noted to be mildly tachycardic and began to complain of cough and dyspnea. At that time, he reported experiencing worsening dyspnea on exertion since his infusion on cycle 2, day 2. Transthoracic echocardiogram revealed a diminished left ventricular EF of 54% (compared with 66%, 22 months prior) and inadequate TR to calculate RV pressure. Pulmonary function tests revealed significantly decreased unadjusted diffusion capacity of the lung, (67% of expected value, compared with 85% of expected value, 19 months prior). The cumulative dose of carfilzomib was 221 mg/m^2^. The patient discontinued carfilzomib and switched treatment to DCEP chemotherapy for two cycles, during which time he achieved a partial response. He then received consolidation treatment with bortezomib, lenalidomide, and prednisone, and 120 days later showed improvement in symptoms; however, exercise tolerance was persistently decreased.

## Discussion

We present these case reports from patients reporting known or suspected cardiac or vascular-related events. Although the exact mechanism of renal damage in Case 3 is not completely clear, the high blood pressure and lack of other symptoms of renal dysfunction suggest that the adverse event was related to the vascular system. The patient’s tolerance of a lower dose of carfilzomib also suggests a potential dose-response relationship with respect to vascular effects. Another patient in our case series developed concomitant acute exacerbation of chronic hypertension while starting carfilzomib treatment and was found to have fibromuscular dysplasia on renal ultrasound, suggesting that treatment-emergent vascular effects may be associated with renal adverse events.

Although the cardiac and vascular-related adverse events presented herein were noted infrequently, they have significant clinical implications, and clinicians should closely monitor at-risk patients (Table 3). At our institution, prior to initiating carfilzomib, we obtain transthoracic echocardiograms for patients with a prior history of anthracycline exposure, age greater than 60 years, known or suspected amyloidosis, or other cardiovascular risk factors. Consistent with data from an epidemiological study on cardiac event rates in patients with MM demonstrating a high prevalence of cardiopulmonary comorbidities [19], it is noteworthy that most patients in our case series who experienced a cardiac toxicity following carfilzomib treatment had a prior history of that adverse event or comorbidity.

**Table 3:** **Recommended monitoring and management of possible carfilzomib**-**associated cardiac and vascular**-**related events**

**(A)** **Prevention of cardiac and vascular-** **related events**	• Monitor cardiopulmonary symptoms
	• Monitor cardiopulmonary signs (vitals, weight, physical exam)
	• Administer carfilzomib over 30 min
	• Check baseline transthoracic echocardiogram, and repeat echocardiogram every two to three cycles^a^ Note, if baseline ejection fraction is below normal, consider utilizing prophylactically strategies listed in (B) Monitor BNP^a^
	• Close follow-up by cardiologist or cardio-oncologist^a^
**(B)** **Management of treatment**-**emergent possibly cardiac and vascular-** **related complications**	• Rule out alternative causes (e.g., pulmonary embolus, pneumonia, anemia)\
	• Hold carfilzomib until toxicity resolves to grade <2.
	• If the decision is made to rechallenge:
	− Implement all preventative/monitoring strategies listed in (A)
	− Consider dose reduction
	− Decrease or eliminate hydration, especially if the patient has completed ≥1 cycle of 27 mg/m^2^ carfilzomib while retaining stable renal function and without experiencing tumor lysis
	− Minimize corticosteroids and concomitant fluid retention
	− Cautious use of diuretics
	− Use of anti-hypertensives as indicated

^a^Recommended for patients who are at increased cardiac risk (e.g., anthracyline exposure, age >60 years, amyloid, coronary artery disease).

BNP, brain natriuretic peptide.

The prescribing information for carfilzomib (Kyprolis®) recommends monitoring patients for cardiac complications and evaluating patients with cardiac imaging and/or other tests as indicated [3]. Given that most of the patients in our case series developed cardiac or vascular-related events relatively soon after starting carfilzomib (i.e., within two cycles), it is likely prudent in patients with cardiac risk factors to monitor the following variables closely during the initial cycles of treatment: cardiopulmonary signs or symptoms (such as dyspnea at rest or with exertion, orthopnea, paroxysmal nocturnal dyspnea, or edema), blood pressure, heart rate, oxygen saturation, daily weight, and viscosity level in patients with high tumor burden (Table 3A). For patients who develop abnormalities of any of these variables (Table 3B), alternative etiologies such as pulmonary embolus, pneumonia, or anemia should be ruled out. The manufacturer of carfilzomib recommends that in instances of pulmonary hypertension, new onset or worsening of CHF, decreased left ventricular function, myocardial ischemia, or grade 3 or 4 cardiac events or pulmonary complications, the carfilzomib dose should be withheld until the event is resolved or returns to baseline. They also recommend that dosing be resumed at the dose used prior to the event or at a reduced dose at the discretion of the physician. We agree that carfilzomib treatment should be withheld, but we suggest that the dose only be resumed upon resolution to below grade 2 and suggest that cardiology consultation and repeat echocardiogram be considered. We also recommend assessment of BNP levels, as a recent retrospective study of patients receiving carfilzomib over 2–10 min found that an elevation of BNP to greater than 400 pg/mL is correlated with increased rates of hospitalization for a cardiovascular complication [20].

Consideration should be given to minimizing concomitant treatments that could contribute to the above symptoms. For example, while administration of intravenous saline (or other appropriate fluid) is recommended as prophylaxis against tumor lysis syndrome, it may be prudent to decrease or eliminate hydration in some patients, as fluid overload may manifest as a severe cardiac or pulmonary event [21]. Such steps should be especially considered when a patient has demonstrated tolerance and stable renal function after one or more cycles of carfilzomib at 27 mg/m^2^ and also, obviously, in the setting of CHF. It may also be beneficial to decrease the dose of steroids in order to minimize fluid retention. As always, symptomatic management such as cautious use of diuretics and initiation of anti-hypertensives as appropriate may be beneficial. Surveillance echocardiograms every two to three cycles should be considered for patients with baseline cardiovascular risk factors. Careful evaluation of blood pressure and antihypertensive medication should also be conducted to reduce risk of renal dysfunction. In patients with renal insufficiency, it is obviously important to determine whether the renal impairment originates from the treatment regimen or from disease progression and to then manage treatment accordingly.

### Potential mechanisms of proteasome inhibition-mediated endothelial dysfunction and heart failure

We hypothesize that the non-hematologic adverse events reported in our case series may stem from proteasome inhibitor–mediated effects on the cardiovascular system, possibly as a result of changes in endothelial nitric oxide synthase (eNOS) activity and nitric oxide (NO) levels. Loss of NO bioavailability leads to impaired vasodilation and endothelial dysfunction [22], and misregulation of NO homeostasis is associated with hypertension, coronary artery disease, CHF, peripheral artery disease, diabetes, and chronic renal failure [23, 24]. Interestingly, the proteasome plays a role in almost every form of eNOS regulation, including its degradation, transcription, and phosphorylation/dephosphorylation [25–28].

*In vitro*, proteasome inhibition seems to modulate eNOS function in a dose-dependent biphasic manner, in which low concentrations of proteasome inhibitors similar to bortezomib improve eNOS function through transcriptional upregulation [29], while at high-dose concentrations, accumulation of ubiquitinated protein phosphatase 2A leads to decreased eNOS activity [28, 30, 31]. *In vivo*, proteasome inhibition with the boronate-based agent MLN-273 in pigs resulted in increased eNOS expression that was uncoupled from NO production and was associated with increased coronary artery oxidative stress and functional and structural changes to the heart consistent with a hypertrophic-restrictive cardiomyopathy phenotype [32, 33]. Similar perturbation of eNOS activity following carfilzomib treatment could potentially result in the toxicities reported in these cases.

It is interesting to note that there are also case reports of bortezomib-treated patients who subsequently developed cardiovascular complications, such as heart failure [34–37], ischemic heart disease [38], and complete heart block [39], and that onset of cardiac symptoms was documented only after each patient reached a cumulative dose of 20.8 mg/m^2^ or greater of bortezomib [34, 35, 37]. While both bortezomib and carfilzomib could theoretically produce endothelial dysfunction through proteasome inhibition–mediated eNOS modulation, the irreversible proteasome inhibition and higher potency of carfilzomib [7] and, perhaps more importantly, the dose-limiting neuropathy associated with bortezomib treatment could potentially explain why the sequelae of endothelial dysfunction may not be observed as frequently with bortezomib treatment.

Given the possible link between carfilzomib treatment and changes in NO homeostasis, the use of NO-releasing agents (such as the nitrate-class drugs nitroglycerin and isosorbide mononitrate or phosphodiesterase inhibitors) may be of interest for treating hypertension in patients receiving carfilzomib. However, further studies are needed to examine this strategy and to investigate whether even longer infusion times (e.g., 60 or 90 min) or use of cardioprotective agents such as the free radical scavenger dexrazoxane may minimize the likelihood of hypertension recurrence.

In our case series, two patients, including Case 4, had baseline cardiomyopathy (CM), likely due to prior anthracyline exposure. In animal models, proteasomal function insufficiency (e.g., increased amounts of ubiquitinated cardiac proteins) has been observed and implicated in the development of ischemic heart disease, hypertrophy, pressure overload, diabetes, and idiopathic dilated CM [40]. This may be due to an increase in the levels of calcineurin and nuclear factors of activated T-cells, which have been associated with maladaptive cardiac hypertrophy following bortezomib-treatment in mouse models [41]. Given these findings, the pathophysiologic changes associated with baseline CM could potentially have played a role in the development of systolic dysfunction after carfilzomib therapy.

The CM cases reported here also suggest that patients with prior anthracycline exposure may have increased susceptibility to cardiotoxicity from reactive oxygen species generated from proteasome inhibitor–induced eNOS uncoupling. However, it should be noted that CHF has also been reported in patients with smoldering MM (grade ≥3, 1/8 patients) or newly diagnosed MM (grade ≥3, 2/18 patients) [42, 43]. Randomized studies are needed to fully understand what impact, if any, factors such as prior treatment have on the incidence of cardiac events following carfilzomib treatment. Serial echocardiograms are being included as a substudy in the phase III study ENDEAVOR (NCT01568866) and will provide important information on the pathophysiology of these events. Also, given that patients 2 and 5 in our series received concomitant antimyeloma agents, the impact of concomitant therapies warrants further investigation as well.

## Conclusions

In conclusion, we report here key cardiac and vascular-related adverse events that were observed in patients with relapsed and/or refractory MM who were treated with carfilzomib. We theorize that these events may be the result of a dose response–dependent perturbation in eNOS activity and NO synthesis that adversely affects cardiac and endothelial cells. Importantly, the patients in this case series who did have treatment-emergent toxicities and did not quickly succumb to disease progression had an improvement or resolution in the toxicity after discontinuation of therapy. In our experience, with appropriate screening (based on cardiovascular risk), monitoring, and symptom management, patients who are at-risk for or who experience a cardiovascular event may still be able to benefit fully from treatment with carfilzomib.

### Consent

Written informed consent was obtained from all patients, or next of kin in the case of deceased patients, for publication in this Case series. Copies of the written consent are available for review by the Editor of this journal.

## Abbreviations

BNP: Brain natriuretic peptide
CCTP: Cyclophosphamide, thalidomide, and prednisone
C_max_: Maximum concentration
CHF: Congestive heart failure
CM: Cardiomyopathy
DCEP: Dexamethasone, cyclophosphamide, etoposide and cisplatin
DS: Durie-Salmon
DVT: Deep vein thrombosis
EF: Ejection fraction
eNOS: Endothelial nitric oxide synthase
Ig: Immunoglobulin
HepB: Hepatitis B
ISS: International staging system
MM: Multiple myeloma
NFAT: Nuclear factors of activated T cells
NO: Nitric oxide
NYHA: New York Heart Association
PFS: Progression-free survival
ORR: Objective response rate
PO: Orally
PRN: As needed
QD: Once-daily
RV: Right ventricular
RVSP: Right ventricular systolic pressure
TR: Tricuspid regurgitation
VDT-(P)ACE: Bortezomib, dexamethasone, thalidomide, cisplatin, doxorubicin, cyclophosphamide, and etoposide.

## References

[1] Kumar SK, Dispenzieri A, Lacy MQ, Gertz MA, Buadi FK, Pandey S, Kapoor P, Dingli D, Hayman SR, Leung N, Lust J, McCurdy A, Russell SJ, Zeldenrust SR, Kyle RA, Rajkumar SV (2014). Continued improvement in survival in multiple myeloma: changes in early mortality and outcomes in older patients. Leukemia.

[2] Siegel DS, Martin T, Wang M, Vij R, Jakubowiak AJ, Lonial S, Trudel S, Kukreti V, Bahlis N, Alsina M, Chanan-Khan A, Buadi F, Reu FJ, Somlo G, Zonder J, Song K, Stewart AK, Stadtmauer E, Kunkel L, Wear S, Wong AF, Orlowski RZ, Jagannath S (2012). A phase 2 study of single-agent carfilzomib (PX-171-003-A1) in patients with relapsed and refractory multiple myeloma. Blood.

[3] Onyx Pharmaceuticals, Inc: *Kyprolis (carfilzomib) package insert*. http://www.kyprolis.com/prescribing-information

[4] Siegel D, Martin T, Nooka A, Harvey RD, Vij R, Niesvizky R, Badros AZ, Jagannath S, McCulloch L, Rajangam K, Lonial S (2013). Integrated safety profile of single-agent carfilzomib: experience from 526 patients enrolled in 4 phase II clinical studies. Haematologica.

[5] Elofsson M, Splittgerber U, Myung J, Mohan R, Crews CM (1999). Towards subunit-specific proteasome inhibitors: synthesis and evaluation of peptide α′, β′-epoxyketones. Chem Biol.

[6] Groettrup M, van den Broek M, Schwarz K, Macagno A, Khan S, de Giuli R, Schmidtke G (2001). Structural plasticity of the proteasome and its function in antigen processing. Crit Rev Immunol.

[7] Demo SD, Kirk CJ, Aujay MA, Buchholz TJ, Dajee M, Ho MN, Jiang J, Laidig GJ, Lewis ER, Parlati F, Shenk KD, Smyth MS, Sun CM, Vallone MK, Woo TM, Molineaux CJ, Bennett MK (2007). Antitumor activity of PR-171, a novel irreversible inhibitor of the proteasome. Cancer Res.

[8] O'Connor OA, Stewart AK, Vallone M, Molineaux CJ, Kunkel LA, Gerecitano JF, Orlowski RZ (2009). A phase 1 dose escalation study of the safety and pharmacokinetics of the novel proteasome inhibitor carfilzomib (PR-171) in patients with hematologic malignancies. Clin Cancer Res.

[9] Wang Z, Yang J, Kirk C, Fang Y, Alsina M, Badros A, Papadopoulos K, Wong A, Woo T, Bomba D, Li J, Infante JR (2013). Clinical pharmacokinetics, metabolism, and drug-drug interaction of carfilzomib. Drug Metab Dispos.

[10] Badros AZ, Vij R, Martin T, Zonder JA, Kunkel L, Wang Z, Lee S, Wong AF, Niesvizky R (2013). Carfilzomib in multiple myeloma patients with renal impairment: pharmacokinetics and safety. Leukemia.

[11] Alsina M, Trudel S, Firman RR, Rosen PJ, O’Connor OA, Comenzo RL, Wong A, Kunkel LA, Molineaux CJ, Goy A (2012). A phase I single-agent study of twice-weekly consecutive-day dosing of the proteasome inhibitor carfilzomib in patients with relapsed or refractory multiple myeloma or lymphoma. Clin Cancer Res.

[12] Jagannath S, Vij R, Stewart AK, Trudel S, Jakubowiak AJ, Reiman T, Somlo G, Bahlis N, Lonial S, Kunkel LA, Wong A, Orlowski RZ, Siegel DS (2012). An open-label single-arm pilot phase II study (PX-171-003-A0) of low-dose, single-agent carfilzomib in patients with relapsed and refractory multiple myeloma. Clin Lymphoma Myeloma Leuk.

[13] Vij R, Wang M, Kaufman JL, Lonial S, Jakubowiak AJ, Stewart AK, Kukreti V, Jagannath S, McDonagh KT, Alsina M, Bahlis NJ, Reu FJ, Gabrail NY, Belch A, Matous JV, Lee P, Rosen P, Sebag M, Vesole DH, Kunkel LA, Wear SM, Wong AF, Orlowski RZ, Siegel DS (2012). An open-label, single-arm, phase 2 (PX-171-004) study of single-agent carfilzomib in bortezomib-naive patients with relapsed and/or refractory multiple myeloma. Blood.

[14] Yang J, Wang Z, Fang Y, Jiang J, Zhao F, Wong H, Bennett MK, Molineaux CJ, Kirk CJ (2011). Pharmacokinetics, pharmacodynamics, metabolism, distribution, and excretion of carfilzomib in rats. Drug Metab Dispos.

[15] Moreau P, Pylypenko H, Grosicki S, Karamanesht I, Leleu X, Grishunina M, Rekhtman G, Masliak Z, Robak T, Shubina A, Arnulf B, Kropff M, Cavet J, Esseltine DL, Feng H, Girgis S, van de Velde H, Deraedt W, Harousseau JL (2011). Subcutaneous versus intravenous administration of bortezomib in patients with relapsed multiple myeloma: a randomised, phase 3, non-inferiority study. Lancet Oncol.

[16] Papadopoulos KP, Siegel DS, Vesole DH, Lee P, Rosen ST, Zojwalla N, Holahan JR, Lee S, Wang Z, Badros A (2014). Phase I study of 30-minute infusion of carfilzomib as single agent or in combination with low-dose dexamethasone in patients with relapsed and/or refractory multiple myeloma. J Clin Oncol.

[17] Lendvai N, Landau H, Lesokhin A, Tsakos I, Koehne G, Chung D, Devlin S, Hassoun H, Giralt SA (2012). Phase II study of infusional carfilzomib in patients with relapsed or refractory multiple myeloma. Blood.

[18] Armenian SH, Sun CL, Vase T, Ness KK, Blum E, Francisco L, Venkataraman K, Samoa R, Wong FL, Forman SJ, Bhatia S (2012). Cardiovascular risk factors in hematopoietic cell transplantation survivors: role in development of subsequent cardiovascular disease. Blood.

[19] Kistler KD, Rajangam K, Faich G, Lanes S (2012). Cardiac event rates in patients with newly diagnosed and relapsed multiple myeloma in US clinical practice. Blood.

[20] Atrash S, Tullos A, Panozzo S, Waheed S, Van Rhee F, Restrepo A, Muzaffar J, Bakhous A, Grazziutti M, Shahid Z, Apewokin S, Abdallah AOA, Barlogie B, Usmani SZ (2013). Retrospective analysis of cardiovascular (CV) events following compassionate use of carfilzomib (CFZ) in patients (Pts) with relapsed and refractory multiple myeloma (RRMM). J Clin Oncol.

[21] Kortuem KM, Stewart AK (2013). Carfilzomib. Blood.

[22] Furchgott RF, Zawadzki J (1980). The obligatory role of endothelial cells in the relaxation of arterial smooth muscle by acetylcholine. Nature.

[23] Endemann DH, Schiffrin E (2004). Endothelial dysfunction. J Am Soc Nephrol.

[24] Fogli S, Nieri P, Breschi MC (2004). The role of nitric oxide in anthracycline toxicity and prospects for pharmacologic prevention of cardiac damage. FASEB J.

[25] Stangl K, Stangl V (2010). The ubiquitin-proteasome pathway and endothelial (dys)function. Cardiovasc Res.

[26] Bender AT, Demady DR, Osawa Y (2000). Ubiquitination of neuronal nitric-oxide synthase in vitro and in vivo. J Biol Chem.

[27] Ito H, Kamei K, Iwamoto I, Inaguma Y, Garcia-Mata R, Sztul E, Kato K (2002). Inhibition of proteasomes induces accumulation, phosphorylation, and recruitment of HSP27 and αB-crystallin to aggresomes. J Biochem.

[28] Wei Q, Xia Y (2006). Proteasome inhibition down-regulates endothelial nitric-oxide synthase phosphorylation and function. J Biol Chem.

[29] Meiners S, Ludwig A, Stangl V, Stangl K (2008). Proteasome inhibitors: poisons and remedies. Med Res Rev.

[30] Meiners S, Ludwig A, Lorenz M, Dreger H, Baumann G, Stangl V, Stangl K (2006). Nontoxic proteasome inhibition activates a protective antioxidant defense response in endothelial cells. Free Radic Biol Med.

[31] Stangl V, Lorenz M, Meiners S, Ludwig A, Bartsch C, Moobed M, Vietzke A, Kinkel HT, Baumann G, Stangl K (2004). Long-term up-regulation of eNOS and improvement of endothelial function by inhibition of the ubiquitin–proteasome pathway. FASEB J.

[32] Herrmann J, Saguner AM, Versari D, Peterson TE, Chade A, Olson M, Lerman LO, Lerman A (2007). Chronic proteasome inhibition contributes to coronary atherosclerosis. Circ Res.

[33] Herrmann J, Wohlert C, Saguner AM, Flores A, Nesbitt LL, Chade A, Lerman LO, Lerman A (2013). Primary proteasome inhibition results in cardiac dysfunction. Eur J Heart Fail.

[34] Gupta A, Pandey A, Sethi S (2012). Bortezomib-induced congestive cardiac failure in a patient with multiple myeloma. Cardiovasc Toxicol.

[35] Honton B, Despas F, Dumonteil N, Rouvellat C, Roussel M, Carrie D, Galinier M, Montastruc JL, Pathak A (2014). Bortezomib and heart failure: case-report and review of the French Pharmacovigilance database. Fundam Clin Pharmacol.

[36] Voortman J, Giaccone G (2006). Severe reversible cardiac failure after bortezomib treatment combined with chemotherapy in a non-small cell lung cancer patient: a case report. BMC Cancer.

[37] Enrico O, Gabriele B, Nadia C, Sara G, Daniele V, Giulia C, Antonio S, Mario P (2007). Unexpected cardiotoxicity in haematological bortezomib treated patients. Br J Haematol.

[38] Takamatsu H, Yamashita T, Kotani T, Sawazaki A, Okumura H, Nakao S (2010). Ischemic heart disease associated with bortezomib treatment combined with dexamethasone in a patient with multiple myeloma. Int J Hematol.

[39] Dasanu CA (2011). Complete heart block secondary to bortezomib use in multiple myeloma. J Oncol Pharm Pract.

[40] Li YF, Wang X (2011). The role of the proteasome in heart disease. Biochim Biophys Acta.

[41] Tang M, Li J, Huang W, Su H, Liang Q, Tian Z, Horak KM, Molkentin D, Wang X (2010). Proteasome functional insufficiency activates the calcineurin-NFAT pathway in cardiomyocytes and promotes maladaptive remodelling of stressed mouse hearts. Cardiovasc Res.

[42] Korde N, Zingone A, Kwok ML, Manasanch EE, Bhutani M, Tageja N, Kazandjian D, Mailankody S, Costello R, Zhang Y, Burton D, Carter G, Wu P, Mulquin M, Zuchlinski D, Maric I, Calvo KR, Braylan RC, Roschewski M, Yuan C, Stetler-Stevenson M, Arthur DC, Lindenberg L, Kurdziel K, Choyke P, Steinberg SM, Landgren O (2013). Phase 2 clinical and correlative study of carfilzomib, lenalidomide, and dexamethasone followed by lenalidomide extended dosing (CRD-R) in newly diagnosed multiple myeloma (MM) patients. Haematologica.

[43] Landgren O, Manasanch EE, Kwok M, Flanders A, Zingone A, Costello R, Mulquin M, Zuchlinski D, Carter G, Maric I, Calvo K, Braylan R, Tembhare P, Yuan C, Stetler-Stevenson M, Arthur DC, Lindenberg L, Choyke P, Kurdziel K, Steinberg S, Roschewski M, Korde N (2013). Clinical and correlative (phase II) pilot study – carfilzomib (CFZ), lenalidomide (LEN), and dexamethasone (Dex) in high risk smoldering multiple myeloma (“early myeloma”). Clin Lymphoma Myeloma Leuk.

[CR44] The pre-publication history for this paper can be accessed here: http://www.biomedcentral.com/1471-2407/14/915/prepub

